# Application of electrospun chitosan-based nanofibers as immobilization matrix for biomolecules

**DOI:** 10.1007/s00253-023-12777-w

**Published:** 2023-09-27

**Authors:** Henrik-Alexander Christ, Nils Peter Daniel, Jennifer Solarczek, Leonard Sebastian Fresenborg, Anett Schallmey, Henning Menzel

**Affiliations:** 1 Institute for Technical Chemistry, Braunschweig University of Technology, Hagenring 30, 38106 Braunschweig, Germany; 2 Institute for Biochemistry, Braunschweig University of Technology, Spielmannstraße 7, 38106 Braunschweig, Germany; 3 Department of Molecular Cell Biology of Plants, Goethe University Frankfurt, Max-von-Laue-Str. 9, 60438 Frankfurt am Main, Germany

**Keywords:** Biotinylated arylazide chitosan, Electrospun nanofibers, Photocrosslinking, Affinity immobilization, Eugenol oxidase

## Abstract

**Abstract:**

Nanofiber meshes from electrospun chitosan, highly modified with biotin and arylazides, are well-suited for application as enzyme immobilization matrices. To test this, catalytically active biomolecules were immobilized onto photocrosslinked nanofibrous nonwovens consisting mainly of biotinylated fungal chitosan and a small amount (10 w%) of poly ethylene oxide. In this study, we show that over 10 μg eugenol oxidase per milligram dry polymer matrix can be loaded on UV-crosslinked chitosan nanofibers. We further demonstrate that bound enzyme activity can be fully retained for over 7 days of storage at ambient conditions in aqueous buffer. Samples loaded at maximum enzyme carrying capacity were tested in a custom-made plug-flow reactor system with online UV-VIS spectroscopy for activity determination. High wettability and durability of the hydrophilic chitosan support matrix enabled continuous oxidation of model substrate vanillyl alcohol into vanillin with constant turnover at flow rates of up to 0.24 L/h for over 6 h. This proves the above hypothesis and enables further application of the fibers as stacked microfluidic membranes, biosensors, or structural starting points for affinity crosslinked enzyme gels.

**Key points:**

**•** *Biotinylated chitosan-based nanofibers retain enzymes via mild affinity interactions*

**•** *Immobilized eugenol oxidase shows high activity and resists continuous washing*

**•** *Nanofiber matrix material tolerated high flow rates in a continuous-flow setup*

**Graphical Abstract:**

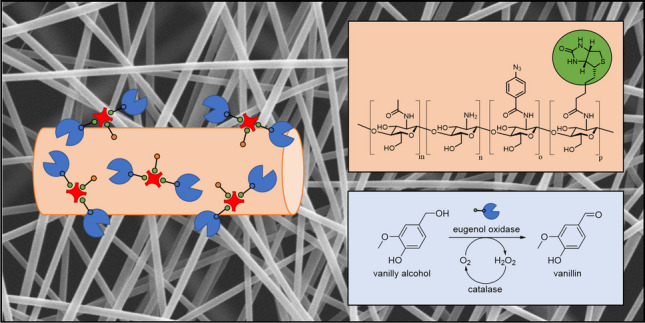

**Supplementary Information:**

The online version contains supplementary material available at 10.1007/s00253-023-12777-w.

## Introduction

The use of enzymes is increasing as they enable mild conditions (e.g., use of aqueous solvent systems and reduced temperatures) for conducting chemical reactions in an efficient and selective way. This leads to safer and more environmentally friendly processes that are highly desired in, for example, medicinal and pharmaceutical applications, food production, or fine chemical manufacturing (Zdarta et al. 2018; Sheldon 2007; Datta et al. 2013; Basso and Serban 2019). Nevertheless, the wide application of enzymes in industrial processes is often hindered by their high production costs and sensitivity against adverse conditions, as well as handling and reusability difficulties (Sheldon 2007; Misson et al. 2015b). These obstacles can be reduced by immobilization of enzymes on solid supports (Zdarta et al. 2018; Sheldon 2007). Furthermore, elaborate processes, such as multi-enzymatic cascades for synthesis of complex bioproducts (Sheldon 2007), active membranes for pollutant removal (Zdarta et al. 2021), biosensor technology (Verma 2017), and medicinally active wound dressings (Zdarta et al. 2018; Sheldon 2007; Zucca and Sanjust 2014), become possible through enzyme immobilization.

In recent years, the technological application of oxygenating enzymes has received particular attention (Lorente-Arevalo et al. 2021). Vanillyl alcohol oxidases such as the eugenol oxidase (EUGO) from *Rhodococcus jostii* are highly interesting candidates as they are oxidases sensu stricto (i.e., accept oxygen as terminal electron acceptor) and able to facilitate oxygenation, methylation, and dehydrogenation of various phenolic compounds with high turnover rates (Fraaije and van Berkel 1997; Nguyen et al. 2016; Jin et al. 2007). A number of valuable products such as vanillin (Kaur and Chakraborty 2013; Chaiyen and Tamanoi 2020; Ewing et al. 2020) or syringaresinol (Habib et al. 2018) have been synthesized using this enzyme family. EUGO is naturally involved in microbial degradation of lignin and has been used in synthetic lignin (Habib et al. 2017) and vanillin (García-Bofill et al. 2021) preparation. Hence, it is a well-suited example to investigate immobilization of enzymes and create useful stationary-phase biocatalysts.

A wide spectrum of support matrices were suggested for immobilization of enzymes (Zdarta et al. 2018; Sheldon 2007; Zucca and Sanjust 2014; Datta et al. 2013; Mohamad et al. 2015; Ribeiro et al. 2021), reaching from inorganic materials (e.g., celite (Ansari and Husain 2012; Khan et al. 2006), zeolites (Díaz and Balkus 1996; Yan et al. 2002), ceramics (Magnan et al. 2004), silica (Petri et al. 2005; Moelans et al. 2005; Borole et al. 2004), activated carbon (Ganesh Kumar et al. 2010), amino functionalized glass beads (Kahraman et al. 2007), charcoal (Rani et al. 2000)) to synthetic organic polymers (e.g., various acrylic copolymer resins such as Eupergit™ C (Kallenberg et al. 2005; Katchalski-Katzir and Kraemer 2000), Sepabeads FP-EP (Mateo et al. 2002), Amberlite XAD-7 (Kirk and Christensen 2002)) to biopolymers derived from nature (e.g., starch (Matto and Husain 2009), cellulose (Al-Adhami et al. 2002; Klein et al. 2011; Namdeo and Bajpai 2009), chitosan (Ribeiro et al. 2021; Vaillant et al. 2000; Kapoor and Kuhad 2007; Chang and Juang 2007; Da S. Moreira et al. 2022; Bösiger et al. 2018; Tighzert et al. 2017; Klein et al. 2013; Rather et al. 2022; Farias et al. 2019; Kouachi 2012), alginate (Matto and Husain 2009; Flores-Maltos et al. 2011; Chen et al. 2011), collagen (Chen et al. 2011; Katwa, L.C., Ramakrishna, M. & Rao, M.R.R. 1981), carrageenan (Rao et al. 2008; Girigowda and Mulimani 2006), Sepharose (Miroliaei 2007)) and even complex biological materials (e.g., fungal mycelium waste streams (Xu et al. 2020) and coconut fibers (Bezerra, Thaís Milena de Souza et al. 2015; Dey et al. 2002)). Likewise, a wide range of chemical processes have been realized using immobilized enzymes, including biodiesel production (Santana et al. 2020), sludge dewatering (Wan et al. 2022), and removal of pollutants from wastewater (Escobedo-Morales et al. 2022). With future applications and requirements in mind, biopolymeric materials have the advantages of being produced from sustainable natural resources and possessing a suitable disposal strategy (Zucca and Sanjust 2014; Ribeiro et al. 2021; Rather et al. 2022).

From the list of biopolymers, the polysaccharide chitosan (CS) is highly promising as its nontoxicity, biocompatibility, and antimicrobial activity are already established (Ribeiro et al. 2021; Ono et al. 2000; Rickett et al. 2011; Hadler et al. 2017; Pellis et al. 2022). It is comprised of *β*-1-4-linked macromolecular chains of *N*-acetyl-glucosamine (GlcNAc) and *N*-glucosamine (GlcN) units (Pellis et al. 2022; Rinaudo 2006; Pillai et al. 2009). This structure is derived from chitin by partial deacetylation of its GlcNAc units (Rinaudo 2006; Pillai et al. 2009). Chitin is highly abundant in the exoskeletons of crustaceans and insects and in the cell walls of filamentous fungi (Pellis et al. 2022; Rinaudo 2006; Ghormade et al. 2017; Hahn et al. 2020). CS from fungal cells is sourced fully renewable and enables an animal-free, cleaner, and ecologically friendlier product with less chemicals and energy intensive production processes (Pellis et al. 2022; Ghormade et al. 2017; Araújo et al. 2019; Dhillon et al. 2013). Protonation of primary amines from GlcN units (pKa ~ 6.3) in acidic media renders CS polycationic (Ribeiro et al. 2021). This results in a natural antimicrobial effect (Farias et al. 2019; Shi et al. 2021; Huan et al. 2020), a high protein binding capability (Rinaudo 2006; Pillai et al. 2009), and a good solubility in aqueous acidic media.

CS was already manufactured into an extensive diversity of forms and geometries (e.g., microspheres (Klein et al. 2013; Klein et al. 2016; Klein et al. 2012), hydrogel disks (Bayramoğlu 2017), nanoparticles (Tighzert et al. 2017; Kouachi 2012; Klein et al. 2012; Banjanac et al. 2016; Betigeri and Neau 2002; Duarte et al. 2017; Misson et al. 2016a), and nanofibers (Tighzert et al. 2017; Misson et al. 2016a; Misson et al. 2016b)) for enzyme immobilization and other applications. From this list, nanofibrous chitosan (CS-NF) sticks out as it combines the material properties of CS with high surface to volume ratio, many interconnected pores, and good mechanical properties. This combination is highly desired in biocatalyst support matrices. Of the methods for CS-NF production (template-assisted extrusion (Raoufi et al. 2016), lyophilization (Tchemtchoua et al. 2011), solution blowing (Liu et al. 2014), centrifugal jet spinning (Upson et al. 2017), and electrospinning (Tighzert et al. 2017; Misson et al. 2016a; Misson et al. 2015a; An et al. 2015)), only the last three result in stable nonwovens consisting of uniform and long nanofibers with sufficient mechanical properties (Li et al. 2015). From these three promising techniques, only electrospinning provides an already scaled up industrial production capacity. Most approaches for electrospinning of chitosan require a small amount of an additive polymer, often poly ethylene oxide (PEO) with high molecular weight, for enhanced spinnability and better fiber properties (Christ and Menzel 2023).

Before application of CS-NF as solid-phase biocatalyst matrices in aqueous media, a treatment for preventing solvation is needed. Different approaches for water stabilization of CS-NF geometries have been described, including reversible stabilization by basic deprotonation (Sangsanoh and Supaphol 2006) or chemical crosslinking with ethylene glycol diglycidyl ether (Liu et al. 2014), glutaraldehyde (Schiffman and Schauer 2007), and genipin (Klein et al. 2016; Li et al. 2015). A safer and more reliable approach is the modification of GlcN units from CS with either acrylates (Romano et al. 2015; Amsden et al. 2007) or photoreactive arylazides (Ono et al. 2000; Rickett et al. 2011; Hadler et al. 2017; Christ et al. 2020; Christ and Menzel 2023) before preparation of the CS-NF immobilization matrix. By irradiation with UV light, these groups can be quickly crosslinked also with spatial control (Ono et al. 2000; Rickett et al. 2011; Hadler et al. 2017; Romano et al. 2015; Amsden et al. 2007; Christ et al. 2020).

A viable biocatalyst matrix must also comprise effective pathways for immobilization of the desired enzymes. Drying as essential step during electrospinning of nanofibers is adverse to the conformational stability of proteins. Therefore, in most cases, the protein needs to be added post-spinning. Covalent immobilization by introducing protein crosslinking functions into the polymer matrix or by using a bifunctional crosslinking reagent (glutaraldehyde (Klein et al. 2016; Klein et al. 2012; Klein et al. 2013b) or genipin (Li et al. 2015)) may cause conformational changes reducing enzymatic activity. Adsorption via monovalent interactions results in much weaker association of protein and solid matrix.

Protein-ligand complexations (affinity immobilization) are a highly specific alternative for affixing functional proteins onto a support matrix. Their modes of binding are less dependent on salt concentration and pH than other methods. Some protein ligand interactions possess high binding enthalpies, sometimes even exceeding those of covalent bonds (avidin-biotin complex, − -854 kJ/mol), while being less harmful to the protein structure than unspecific covalent modification methods (General et al. 2012). For these reasons, affinity chromatography based on ligand-binding polypeptides (e.g., poly histidine, glutathione-S-esterase, Strep™-peptide, various antibody antigens) has become a dominant technique in protein isolation. Especially, the interaction of avidin, streptavidin, and derived proteins (such as StrepTactin™) with biotin or the synthetically generated Strep-peptides (StrepTag-II™) is an interesting option for immobilization of proteins to CS matrices. These proteins occur as homotetramers with four binding sites for biotin/Strep-peptide and enable the formation of a “bridge” between a StrepTag™-bearing protein and a biotinylated matrix (Maertens et al. 2015; Lorsch 2015; Schmidt and Skerra 2015).

In previous studies, methods for efficient incorporation of 4-azidobenzoic acid (Az, for subsequent UV-induced crosslinking) and biotin (Bio, for protein affinity immobilization) into CS (CS-Az-Bio) were demonstrated (Hadler et al. 2017; Christ et al. 2021). Recently, some of us reported an optimized electrospinning and photocrosslinking process for generation of nanofiber materials based on these highly modified derivatives and PEO (Christ and Menzel 2023). Unlike other approaches, our method reliably yields fully water-stable nanofiber mats (CS-Az-Bio-NF). Here, we report the successful immobilization of a recombinant 6xHis-eugenol-oxidase-StrepTag-II™ from *Rhodococcus jostii* on photocrosslinked CS-Az-Bio-NF. Immobilization was achieved via both unspecific interaction with fiber surface and complexation of covalently linked biotinyl residues by an EUGO-StrepTactin™ complex. Furthermore, mats were used for oxidation of vanillyl alcohol to vanillin in a continuous-flow system, demonstrating their applicability for creation of highly effective solid-phase biocatalysts.

## Materials and methods

### Chemicals

Sodium hydroxide (NaOH, > 99%), Tris–Base (> 99%), KH_2_PO_4_ (99%), K_2_HPO_4_ (> 99%), isopropyl-β-D-thiogalactopyranoside (> 99%), 4-hydroxy-3-methoxybenzaldehyde (vanillin, 99%), 4-(hydroxymethyl)-2-methoxyphenol (vanillyl alcohol, 99%), and sodium chloride (NaCl, > 99%) were obtained from Fisher Scientific UK Limited (Loughborough, UK). Glycerol (99.5%), glycine (biotechnological grade), Na_2_SO_4_ (> 99%), and peptone from casein as well as yeast extract (both in purity for microbiology) were used as obtained from VWR International (Radnor, Pennsylvania, USA). Imidazole (99%), kanamycin sulfate (> 99%), and flavin adenine dinucleotide (FAD, > 95%) were purchased from Roth GmbH + Co. KG (Karlsruhe, Germany). Crude fungal chitosan was obtained from KitoZyme (KiOnutrime-CsH, Herstal, Liege, Belgium) and modified by ourselves according to a previously published method (Christ et al. 2021). StrepTactin™ was supplied from IBA Lifesciences GmbH (Göttingen, Germany) while catalase (from *Corynebacterium glutamicum*, 500,000 U/mL), poly ethylene oxide (PEO, Mw = 9,000,000 g mol^−1^), and lysozyme (from chicken egg, > 99%) were purchased from Merck (Darmstadt, Germany). All enzymes were rehydrated in filtrated sterile Millipore water before use.

### Preparation of electrospun immobilization membranes from biotinylated chitosan

Electrospun nonwovens studied here were comprised of fungal CS and a small amount (10 w% of solid content in spinning solution) of PEO. The purified polysaccharide was modified before spinning at the primary amine group of the repetitive unit. By using optimized carbodiimide chemistry according to a previously published method of ours (Christ et al. 2021), we were able to modify CS with 4-azidobenzoic acid (Az) and biotin (Bio) in one batch with maximal coupling efficiency and yield. The resulting CS-Az-Bio derivative was obtained modified with 16% of each, Az and Bio, relating to all repetitive units of CS. An exemplary ATR-IR spectrum of the specific derivative used for this study can be found in Fig. S4. More detailed information for this method can be found elsewhere (Christ et al. 2021 and Christ and Menzel 2023). Production of nanofibers from CS-Az-Bio and PEO (CS-Az-Bio-NF) was accomplished with a custom-made electrospinning instrument. This apparatus, as well as the specific conditions and formulation for successful electrospinning, is described in more detail elsewhere (Christ and Menzel 2023). An exemplary ATR-IR spectrum (see Fig. S4) and two SEM images (see Fig. S5) of the electrospun and photocrosslinked CS-Az-Bio-NF used in this study are given in the supporting information. The finished membranes could be stored over long periods of time for more than 6 months at ambient laboratory conditions in the dark without detectable changes in functionality. Additionally, CS nanofiber meshes from chitosan with 16% of all its saccharide repeating units modified with Az were produced using the same methods (CS-Az-NF). These fibers did not contain any biotinyl residues and were therefore used as a control in the following experiments. In order to secure consistent sample sizes, round discs with 15 mm diameter were punched out of all electrospun fiber meshes, peeled off from the support structure, and weighted thoroughly before use (1.5 ± 0.5 mg/sample disc).

### Photocrosslinking and preparation of samples for further experiments

Two different irradiation conditions were tested for comparison during photocrosslinking of all samples. Most samples were irradiated after electrospinning, using UV light at 290 +/− 50 nm at an irradiance of 100 mW/cm^2^ for 10, 60, or 180 s. These samples were individually prepared by irradiation with UV light from a Hg lamp (350 W, model 68810, L.O.T-Oriel Holding AG, Darmstadt, Germany), an optical filter (UG11 glass, 50·50·5 mm, Schott AG, Mainz, Germany), and a water filter for excluding undesired wavelengths. All other samples were subjected to light within a spectral range of 200–600 nm at an irradiance of 25 mW/cm^2^, shone directly onto the collector during electrospinning. The light source used in this case was a HP-120i point light source integrated into the electrospinning process via a quartz wave guide (both from Opsytec Dr. Groebel, Ettlingen, Germany). The latter method was beneficial for high-throughput generation of samples as a complete fiber mesh was crosslinked during spinning. However, it bears higher risk of decreasing the membrane functionality by radiation damage of polymer chains or integral functional groups such as biotin. This is especially true when long electrospinning processes and thus long irradiation periods such as done here for 240 min are performed. As highly different crosslinking methods were tested in this work, it was expected that the treatment would potentially also damage a certain amount of polymer chains and lead to release of unbound CS-Az-Bio fragments. These biotinylated chains could potentially intercept the A4E4 complex and form nonimmobilized particles that would interfere with a full immobilization. To check and account for this phenomenon, fiber samples irradiated and crosslinked for different amounts of time were subjected to three consecutive washing steps for 10/10/30 min in 1 mL main buffer each. A NaOH·glycine solution (50 mM, pH 8) was used as main buffer system for all experiments, beside isolation and preparation of EUGO. UV-VIS spectra of resulting supernatants from this experiment were taken and the absorbance at 273 nm was determined as this would point towards a release of CS-Az-Bio into the tested solution.

### Cloning, expression, and purification of EUGO

Vector pEUGOA was a kind gift from Prof. Marco Fraaije (University of Groningen, the Netherlands). The gene of EUGO from *Rhodococcus* sp. RHA1 was amplified by PCR from vector pEUGOA (Jin et al. 2007) using primers Fw_pBAD: 5′-CGGCGTCACACTTTGCTATGC-3′ and Rv_eugo-STII_HindIII: 5′-TATGTCAA-GCTTATTTCTCGAACTGCGGGTGGCTCCAAGCGGAGAGGTTTTGGCCACGGAACC-3′. Thereby, the sequence encoding the Strep-tag^®^II (IBA Lifesciences GmbH) was added at the 3′ end of the EUGO gene and restriction site *Hin*dIII (underlined) was introduced. The EUGO-Strep-tag^®^II gene was subsequently cloned into vector pET-28a(+) using restriction enzymes *Nde*I and *Hin*dIII. The resulting vector was named pET-28-eugo-STII. Expression of the EUGO-Strep-tag^®^II gene from vector pET-28-eugo-STII, utilizing a T7 promotor, resulted in N-terminal hexahistidin (His_6_)-tag fusion for simple one-step purification and C-terminal Strep-tag^®^II fusion for later immobilization.

EUGO was heterologously produced in 150 mL scale in *Escherichia coli* BL21 (DE3) gold cells. For this, 150 mL TB medium (4 mL L^−1^ glycerol, 12 g L^−1^ peptone, 24 g L^−1^ yeast extract, 0.17 M KH_2_PO_4_, 0.74 M K_2_HPO_4_) supplemented with 50 mg L^−1^ kanamycin was inoculated with 1% of a respective overnight culture and cultivated at 37 °C and 200 rpm until an OD_600_ between 0.6 and 0.8 was reached. Then, the culture was induced with 0.2 mM isopropyl-β-D-thiogalactopyranoside and cultivated for another 19 h at 30 °C and 200 rpm. After expression, cells were harvested by centrifugation (3488 g, 20 min, 4 °C) and cell pellets were stored at − 20 °C until further use.

Protein purification from cell pellets was performed via immobilized metal ion affinity chromatography using a gravity flow column containing 2 mL Ni Sepharose™ 6 Fast Flow resin (Cytiva, Marlborough, MA, USA). Frozen cell pellets were thawed on ice and re-suspended in 10 mL lysis buffer (binding buffer (50 mM Tris/SO_4_, 25 mM imidazole, 300 mM Na_2_SO_4_, pH 7.9) + 1 mg mL^−1^ lysozyme and 0.3 mg mL^−1^). The cells were disrupted by sonication (5 min, 65% amplitude, cycles of 5 s pulse and 10 s pause), and cell debris was removed by centrifugation (18,533 g, 45 min, 4 °C). After filtration through a 0.45-μm cellulose acetate membrane filter, the resulting cell-free extract (CFE) was loaded on the equilibrated column with 5 column volumes of binding buffer. Elution of the His-tagged protein was performed with 15 mL of elution buffer (50 mM Tris/SO_4_, 400 mM imidazole, 300 mM Na_2_SO_4_, pH 7.9), and the protein-containing fractions were identified by yellow color. These fractions were pooled and concentrated via ultrafiltration using a Vivaspin® Turbo 15 Ultrafiltration unit (Sartorius, Göttingen, Germany) to a volume of 2.5 mL. Desalting was performed according to the manufacturer’s instructions using a PD-10 column from Cytiva while the protein solution in storage buffer (50 mm Tris/SO_4_, pH 8, 10% (v/v) glycerol) was stored in 200 μL aliquots at − 20 °C until further use.

### Investigation of EUGO/StrepTactin™ complex formation

Recombinant EUGO carrying a C-terminal StrepTag-II™ was incubated with StrepTactin™ in different molar ratios for 18 h in main buffer (a NaOH·glycine solution (50 mM, pH 8)) to investigate the formation of complexes. In several batches, StrepTactin™ was mixed in a concentration of 75.0 μM with 37.5 μM EUGO (molar ratio StrepTactin™/EUGO = 2:1), 75.0 μM EUGO (1:1), 150 μM EUGO (1:2), and 750 μM EUGO (1:10). Complex formation was subsequently analyzed by size exclusion chromatography using the ÄKTA Pure system with a Superdex 200 10/300 GL column from Cytiva according to the manufacturer’s instructions. The sample was loaded onto the column in a volume of 100 μL and separation was performed at a flow rate of 0.5 mL/min using 50 mM Tris–Base (pH 8) containing 150 mM NaCl as running buffer.

A protein standard mix (product number 69385, 15–600 kDa for testing SEC/GFC columns) from Merck (Darmstadt, Germany) was used for size calibration of the applied ÄKTA system and the estimation of molecular weights of analyzed protein complexes. For an image of the elugram of that protein standard mix, refer to Fig. S1. A hyperbole fit: ($f(x)=\frac{524.4}{x+39.6}+9.5$ , *χ*^2^ = 0.797, *R*^2^ = 0.992), was used to estimate analyte sizes from their respective elution volumes.

Protein electrophoresis (SDS-PAGE) of relevant fractions from ÄKTA elution was performed using custom-made electrophoresis gels containing 12% acrylamide. Pierce™ unstained protein MW marker (product number 26610, a mixture of seven native proteins in a size region of 14.4 to 116 kDa) from Thermo Fisher Scientific (Braunschweig, Germany) was used as size standard (protein ladder) for this method.

### Activity determination of free EUGO

The activity of EUGO in solution was measured, determining the initial reaction rate of the proteins (v_0_-determination) in vanillin assays as follows: a certain amount of supernatant or A4E4 complex, 250 μL if not stated otherwise, was added to a quartz glass cuvette containing a respective amount of main buffer with a final volume of 600 μL. After this, the absorbance of the solution at 340 nm was spectrophotometrically measured over a time course of 120 s with a resolution of 0.5 s^−1^. After a waiting period of 30 s to generate a baseline, 400 μL of 2 mM vanillyl alcohol in main buffer was added and the solution was quickly stirred. The slope of the curve resulting from the first 15 s after substrate addition was derived using a linear fit by the measurement device internal method in kinetic modus. A V-630 UV-VIS spectrophotometer from Jasco (Tokyo, Japan) was used for all spectrophotometric measurements.

### Immobilization experiments

All samples used for immobilization experiments (dry weight of 1.5 ± 0.5 mg/sample disc) were hydrated and washed three times for 10 min in 1 mL main buffer each before usage to remove any unbound CS-Az-Bio chain fragments. Blocked samples were additionally incubated for 90 min in 1 mL blocking buffer (1 w/v% skimmed milk powder + 0,041 μM biotin in main buffer, pH 8) and washed again three times for 10 min in 1 mL main buffer. For immobilization of EUGO, the samples were subsequently incubated for 23 h in 1 mL main buffer containing different amounts of A4E4 complex. The resulting supernatant was subjected to activity assays to determine the loss of enzyme activity resulting from contact with fiber samples. Three washing steps for 10 min in 1 mL main buffer were performed to rid the samples of unbound enzyme residues. After this, the fiber samples were tested for immobilized activity. All washing and incubation steps were performed under mild agitation conditions at 25 °C. All experiments were performed in triplicate.

### Activity assay of fiber samples

The standard procedure for determination of bound enzyme activity was performed by incubation of a fiber sample for 120 s in main buffer containing 800 μM vanillyl alcohol as chromogenic substrate at 25 °C and vivid agitation. The assay was stopped by manually removing and washing the fiber sample. The activity of a given sample was determined by measuring spectrophotometrically the absorbance at 340 nm of produced vanillin in the supernatant. The specific activity per fiber mass was calculated using an external calibration of vanillin and dry sample weight.

### Washing experiments

The stability of protein binding was tested by subjecting fiber samples loaded with EUGO to different washing buffers. In between these experiments, the samples were always kept in main buffer at ambient conditions. Activity assays for determination of bound enzyme activity were performed regularly for the initial 7 days after immobilization without changing the buffer. After this period, 1 mL high-salt buffer (main buffer + 400 mM NaCl, pH 8) was used per sample and was changed always after performing an activity assay. At day 16, high-salt buffer containing excess of biotin was used. To account for systematic variations, between sets of separately measured samples, only the relative activity was compared and discussed here. The supernatant of days 7, 8, and 9 was additionally subjected to activity assays for determination of unbound EUGO, in order to check for EUGO that was detached from fibers during the washing step.

### Continuous vanillin production—proof of concept

Fiber samples from previous experiments were also used for continuous production of vanillin in order to show proof of concept for a potential application of the described system. For this, a fiber sample loaded with A4E4 complex was fixed inside a reusable poly propylene syringe filter with a membrane diameter of 13 mm (membrane area = 133 mm^2^) from Finetech (New Taipei City, Taiwan). This small flow-through reactor cell was attached to a 20-mL syringe, driven by a syringe pump LA-30 from HLL Landgraf (Langenhagen, Germany). The output of the reactor was led through PTFE tubing through a Type 58 Macro flow through quartz glass cuvette from FireflySci Inc. (Northport, NY, USA) with a volume of 2.520 mL inside a UV-VIS spectrophotometer and finally into a waste container. (For an image of the setup, refer to Fig. S2.) With this setup, it was possible to determine the fiber mesh activity at any time point by spectrophotometrically measuring the absorption of vanillin as a signal at 340 nm in the cuvette. All experiments were performed at a constant flow rate at ambient conditions and in main buffer at pH 8.0. Relevant factors varied between experiments were the amount of substrate vanillyl alcohol (0–2000 μM) and the flow rate (0.25–4.0 mL/min) as well as the addition of excess catalase into the buffer system. An experiment was always started by flow through of 10 mL main buffer, followed by 20 mL substrate in buffer, and ended with a rinsing step of 10 mL main buffer without substrate. The resulting data clearly shows main buffer phase, dead volume, mixing kinetic of the cuvette, and stable plateau of enzyme activity, as well as reverse mixing kinetic of the cuvette when changing back to main buffer. An example of the raw data including these sections is given in Fig. 4. Only the stable plateau phase was used for determination of immobilized EUGO activity, neglecting cuvette mixing as well as main buffer phases.

### Statistical analysis

Null hypothesis testing of scalar measurements was performed using two-tailed unpaired two-sample *t*-test (for comparison of two samples) or one-way ANOVA (*α* = 0.05, for comparison of more than two samples) followed by pairwise comparison with the Tukey-HSD test (*α* = 0.05). All statistical analysis was performed using Microsoft Excel (Microsoft Office Home and Student 2019).

## Results

**Fig. 1:**
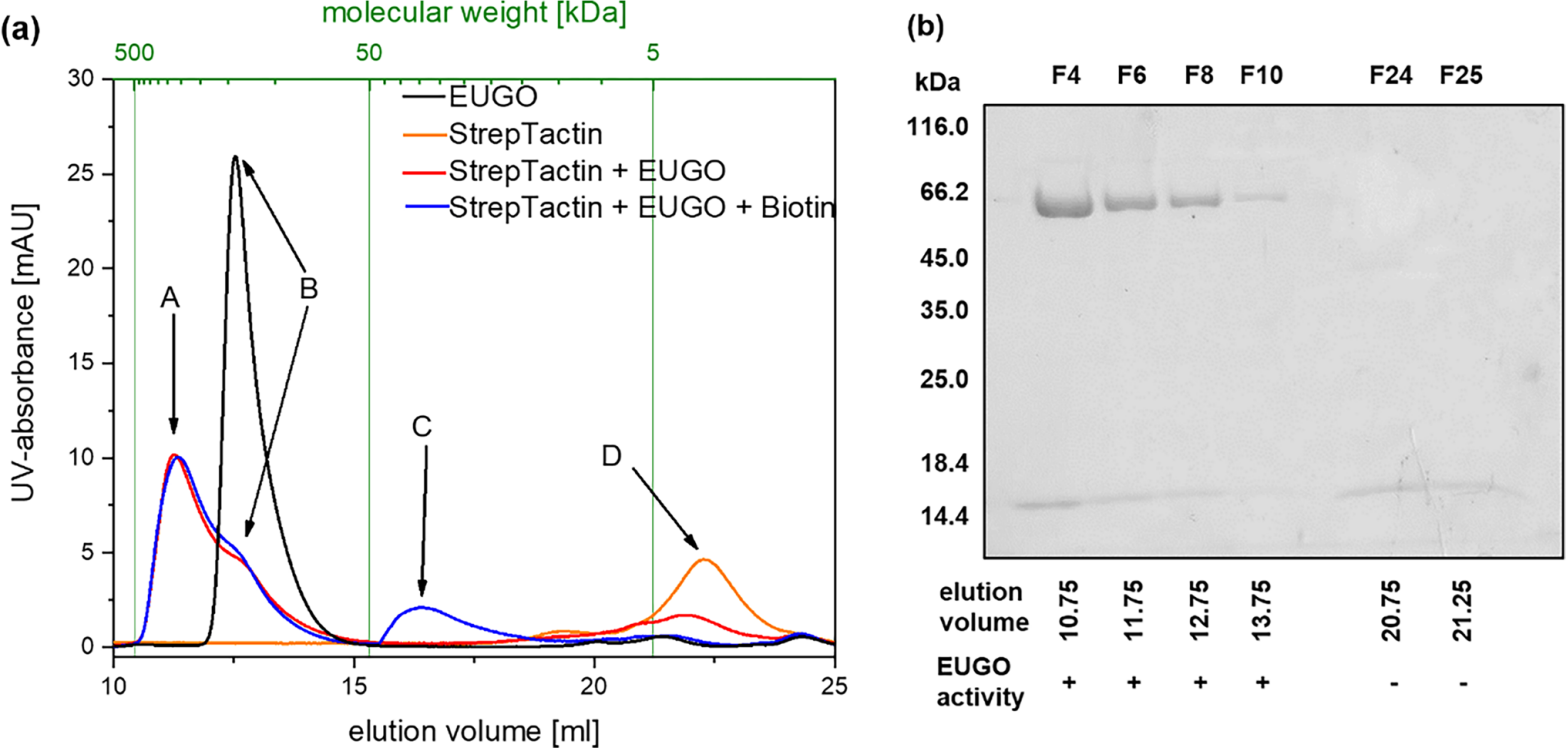
**a** Stacked elugrams of pure EUGO (black) and StrepTactin™ (orange). Also shown are two elugrams of both proteins incubated together in a 1:1 ratio for 18 h with (blue) and without (red) an excess of biotin in the buffer system. Peaks marked with letters represent formed EUGO/StrepTactin™-complex (A4E4, A), pure or unreacted EUGO (B), StrepTactin™ in the presence of biotin (C), and pure StrepTactin™ (D). **b** SDS-PAGE from fractions collected during elution of A4E4 complex (F4, F6, F8, F10, taken from the sample corresponding to the red line) is showing both EUGO and StrepTactin™ monomers. Additionally, EUGO activity could be determined in those fractions by standard vanillin assay. Fractions F24 and F25 show no EUGO activity as only StrepTactin™ was present in these fractions, as can also be observed in SDS-PAGE results

In order to investigate the ability of biotinylated CS-NF mats to immobilize enzymes in a catalytically active form, a complex of 6xHis-eugenol-oxidase-StrepTag-II™ (EUGO) and StrepTactin™ was used as a model system. To investigate complex formation, both proteins were incubated in different molar ratios and subsequently analyzed via size exclusion chromatography. The 1:1 ratio, related to the monomeric subunits of EUGO and StrepTactin™, is shown in Fig. 1 a. All other ratios are shown in Fig. S3.

Pure EUGO was eluted at a volume of 12.53 mL (Fig. 1 a, peak B) corresponding to an estimated molecular weight of 130 ± 50 kDa. The mixture of EUGO and StrepTactin™ resulted in a main signal at a lower elution volume (Fig. 1 a, peak A, 11.25 mL, 250 ± 80 kDa) with a shoulder at the volume corresponding to unbound EUGO (Fig. 1 a, peak B). Also, a second signal at 22.25 mL elution volume (Fig. 1 a, peak D) was found. When the protein mixture was co-incubated together with an excess of biotin, signal D was found shifted into higher elution volume regions (Fig. 1 a, peak C, 16.26 mL, 40 ± 20 kDa). Fractions of the main signal showed high specific EUGO activity in vanillin assays. Moreover, when these fractions were analyzed via SDS-PAGE, two bands in weight regions corresponding to EUGO and StrepTactin™ monomers were found (Fig. 1 b).

**Fig. 2:**
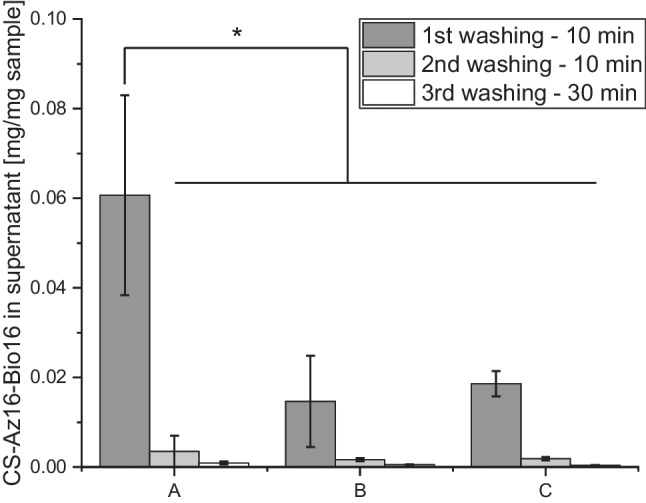
Results from the three washing steps of CS-Az-Bio-NF mats before immobilization of target enzyme complex. Absorbance of supernatant at 273 nm indicates release of CS-Az-Bio chains or fragments thereof. Amount of released CS-Az-Bio depends on irradiation times: A = 10 s (1 J/cm^2^), B = 60 s (6 J/cm^2^), and C = 180 s (18 J/cm^2^). Null hypothesis testing for zero difference between all samples was performed with ANOVA (*α* = 0.05) followed by pairwise comparison with the Tukey-HSD test

Before use of the nanofibers for immobilization of proteins, two treatments, photocrosslinking and initial soaking, needed to be carried out. Irradiation with UV light causes the Az side chains to lose N_2_ and form highly reactive nitrenes that attack C-H or N-H groups of neighboring polymer chains, thereby forming a tightly linked network (Christ and Menzel 2023). At a degree of substitution (DS) of over 15% of Az, this treatment is sufficient to render the mats long-term water stable even at acidic conditions (1 M HCl) (Christ and Menzel 2023). However, in preliminary experiments, we observed precipitation (turbidity visible via eye) in solutions containing EUGO and StrepTactin™ as soon as dry, crosslinked CS-Az-NF mats were added. We hypothesize that either unbound CS-Az-Bio chains or fragments thereof were dissolved and subsequently co-precipitated with the proteins present. This could happen either by nonspecific interactions or by affinity interactions between StrepTactin™ and biotinyl residues of CS-Az-Bio. Indeed, when freshly photocrosslinked mats were transferred to aqueous washing buffer, an absorbance signal with a maximum at 273 nm indicated the presence of dissolved CS-Az-Bio in the supernatant. The amount of CS-Az-Bio in solution was estimated via external calibration with 4-azidobenzoic acid and data from determining the of degree of substitution (DS_Az_ and DS_Bio_) and the degree of acetylation (DA), stemming from ^1^H-NMR spectra of the pure polymers. This was done under the assumption that the dissolved polymer chains or fragments would contain similar amounts of substituents than the untreated polymers and that calibration with 4-azidobenzoic acid is sufficient for estimation of CS-Az-Bio. The amounts of leached CS-Az-Bio chains from fibers samples (*N* = 3) photocrosslinked at different conditions and dissolved in the supernatants of three sequential washing steps are summarized in Fig. 2.

Increasing the irradiation time from 10 s (Fig. 2, A) to 60 s or higher (Fig. 2, B and C) resulted in a significant reduction of dissolved polymer in the supernatant. The same was achieved with repeated washing. After removal of the leached polymer chains, the mats remained stable and did not release more material. Thus, for following experiments, all samples were crosslinked for > 60 s and washed 3 times in main buffer before use.

To test the ability of biotinylated CS-NF mats to bind the protein complexes, crosslinked and pre-washed CS-Az-Bio-NF samples, as well as similarly treated control samples without biotin (CS-Az-NF), were incubated in 1 mL of protein solution (concentration of 144.4 μg/mL EUGO-dimers and 60 μg/mL StrepTactin™-tetramers) overnight. After washing and incubation for 1 day in buffer, catalytic activity of the CS-Az-Bio-NF samples (19.4 ± 0.9 μM/min·mg fibers) was significantly (*p* = 0.02) increased compared to CS-Az-NF samples (14.6 ± 1.9 μM/min·mg). In order to separate the specific from the nonspecific immobilization effect, one set of both CS-Az-Bio-NF and CS-Az-NF samples was incubated in skimmed milk (1% dry mass) prior to A4E4 application to saturate the unspecific protein binding capacity of the mats (blocked samples). From these two sets, biotinylated samples (CS-Az-Bio-NF) retained most of their binding ability after blocking and incubation for 1 day in buffer (activity: 15.2 ± 1.1 μM/min·mg), while biotin-free CS-Az-NF samples showed a significantly (*p* = 0.0002) lower EUGO activity at comparable conditions (5.1 ± 0.8 μM/min·mg).

**Fig. 3:**
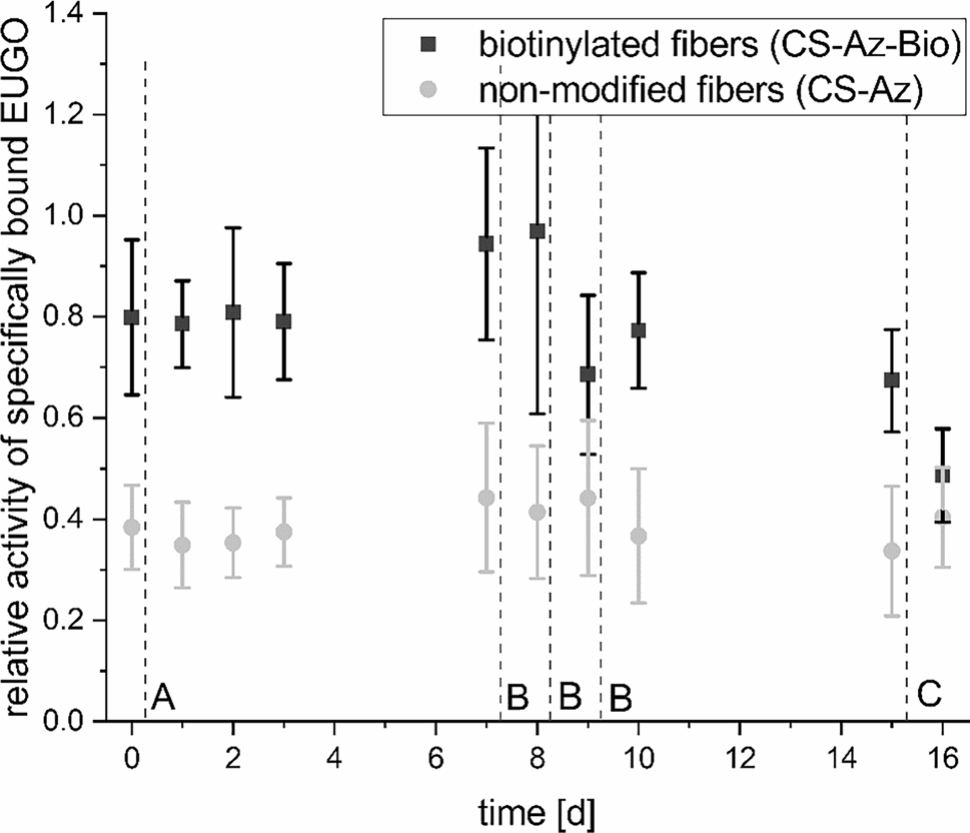
Activity of specifically bound EUGO from blocked fiber samples relative to untreated samples. Letters A–C indicate each time incubation buffer was exchanged: main buffer (A), high salt buffer without (B), and with biotin (C). The original data was also analyzed with ANOVA, demonstrating that the untreated CS-Az-NF meshes showed significantly lower activity in buffer A compared to both the biotinylated and blocked samples (see Fig. S6)

For practical application, a high protein retention of the immobilization matrix over time and under varying conditions is essential. The A4E4-loaded mats were transferred to 1 mL main buffer (pH 8) and stored under mild agitation and ambient laboratory conditions for 7 days. Neither CS-Az-Bio-NF nor CS-Az-NF mats showed any significant change in relative enzymatic activity over this period of time (Fig. 3, line A). Also, no EUGO activity was observed in the supernatant of the samples after 7 days. To investigate the resistance of the binding further, the samples were subsequently transferred to high salt (400 mM) buffer and washed every day for 3 days in a row (Fig. 3, line B). The decrease in relative activity under these high salt conditions was 7-fold higher for CS-Az-Bio-NF compared to CS-Az-NF. After 15 days, an excess amount of biotin was added to the samples, expected to cause dissociation of remaining biotin-bound StrepTactin™ from the CS-Az-Bio-NF matrix. Indeed, relative EUGO activity of CS-Az-Bio-NF mats dropped to the level of CS-Az-NF mats, while remaining unchanged in the latter, indicating a replacement of StrepTag-II™ and thus a release of EUGO by free biotin. As less than 10% of the applied 144.4 μg EUGO per sample were bound by the mats, A4E4 complex concentration per mat was lowered to 28.8 μg (in respect to EUGO-dimer) in all further experiments.

Above results were produced, using CS-Az-Bio-NF samples that were treated by irradiation with UV light at 100 mW/cm^2^ for 180 s, resulting in a total energy flux of 18 J/cm^2^. It has been observed before that biotin is, at least moderately, susceptible to degradation via UV light of that energy (Islam et al. 2016). To investigate whether photocrosslinking influences the immobilization capacity of the studied fibers, we compared the binding capacity of CS-Az-Bio mats subjected to different UV-treatments (0 s and 180 s, constant energy flux (0 and 18 J/cm^2^), at narrow spectrum). Additionally, a set of CS-Az-Bio and CS-Az fibers as control were used that were irradiated in continuous mode (240 min (14400 s) at full UV spectrum at 25 mW/cm^2^ (360 J/cm^2^)). All samples were subsequently incubated in buffer containing StrepTactin™ and EUGO in a 1:1 ratio (28.8 μg EUGO per sample, sample weight ~ 1 mg) overnight. Remaining activity of the resulting supernatant was measured using v_0_-determination in vanillin assay. These values, compared with the original activity of the A4E4 complex solution, were used to calculate the amount of EUGO adsorbed on the fiber meshes (Table 1). The immobilized EUGO activity was also measured using an end-point-determination in standard vanillin assay. The results were used to calculate the amount of EUGO immobilized on the samples, assuming a *k*_cat_ of free A4E4 complex of 4 s^−1^ (Table 1).

**Table 1:** Degradation of binding capacity of biotinylated chitosan nanofibers by UV irradiation. Null hypothesis testing for zero difference between all samples was performed with ANOVA (*α* = 0.05) followed by pairwise comparison which was done with the Tukey-HSD test

Chitosan derivative used	Crosslinking time	Irradiation energy	Activity loss in supernatant†		EUGO adsorbed, determined from activity loss in supernatant	Immobilized activity on fiber sample		EUGO adsorbed, determined from immobilized activity on fiber sample
	(min)	(J/cm^2^)	(μM/min*mg)^‡,*^		(μg/mg)^‡^	(μM/min*mg) ^‡,*^		(μg/mg)^‡^
CS-Az-Bio	0	0	110 ± 20	a	21 ± 3	3.7 ± 0.7	a	1.0 ± 0.2
CS-Az-Bio	3	18	70 ± 10	b	13 ± 2	17.9 ± 0.7	b	4.6 ± 0.2
CS-Az-Bio	240	360	24 ± 2	c	5.0 ± 0.4	3.7 ± 0.8	a	1.0 ± 0.2
CS-Az	240	360	55 ± 4	b	10.9 ± 0.8	5.1 ±- 0.2	a	1.32 ± 0.05

^†^28.8 μg was applied per sample

^‡^values are calculated in respect to the mass of each individual fiber sample

*The two sets of data were tested for significance separately from each other using ANOVA and pairwise comparison with the Tukey-HSD test, as they were generated using different methodologies. Letter code from a to c was used to group data into categories with significant differences

**Fig. 4:**
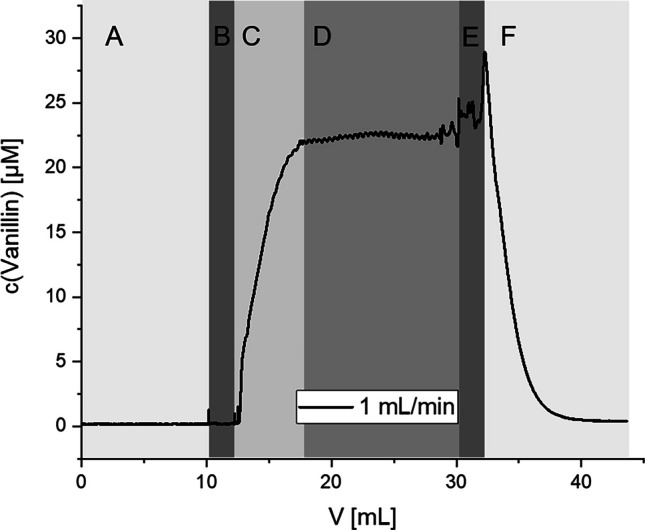
Exemplary reading from continuous-flow reactor setup with all observable phases: initial purging of the system with buffer (A), start of reaction with substrate buffer (B–D) and final purging with buffer (E–F). Dead volumes (B and E) as well as cuvette equilibration phase (C and F) and plateau phase (D) are also visible. Periodic fluctuation visible at D results from rotational movement of syringe pump driver

For many potential applications of protein-loaded CS-NF (e.g., flow cells, fermenters, in vivo tissue engineering), the matrix has to maintain its properties under continuous-flow conditions. A continuous-flow demonstrator (see Fig. S2) was built by fixing a mat in a small cell connected to a syringe pump and a UV-VIS flow-through cuvette with silicone tubes. This enabled online photometric determination of the product concentration (see Fig. 4) at controlled flow rates.

The sample used for generation of the results shown in Fig. 4 had a dry mass of 1.303 mg nanofibers and an immobilized EUGO activity of 18.6 μM/min·mg (activity of complete sample = 24.3 μM/min). Before each measurement, the system was purged with 10 mL wash buffer (Fig. 4, A). After start of substrate application and passage of 2.2 mL dead volume (tubing volume, Fig. 4, B), a hyperbolic increase of vanillin concentration was observed (C), representing the replacement of wash buffer from the large (2.5 mL) cuvette volume. The signal stabilized after 6.2 mL (D). The periodic fluctuation of the stable plateau can be ascribed to flow-rate fluctuations as the frequency matched the revolution number of the syringe pump driver. After completing the measurement, the flow had to be interrupted for a manual change back to wash buffer (F), resulting in a brief increase in vanillin concentration (E) while substrate buffer remained stationary on the mat. The averaged signal after stabilization corresponds to an activity of 22.5 ± 0.2 μM/min. In none of our experiments did we observe a reduction in EUGO activity over time.

The continuous-flow setup also allowed for accurate determination of kinetic behavior of the immobilized complex. Here, higher *K*_M_ and lower *k*_cat_ values compared to nonimmobilized EUGO were observed. Moreover, *K*_M_ was found to double when increasing the flow rate from 1 to 2 mL/min (Table 2).

**Table 2:** Comparison of Michaelis-Menten kinetics from EUGO in different environments in main buffer: free EUGO, bound in complex with StrepTactin™ and immobilized on CS-Az-Bio-NF membrane

Environment of EUGO	*K*_M_ (μM)	*k*_cat_ (s^−1^)	Experimental method	Experimental conditions
Free EUGO from literature (Jin et al. 2007)	40	12	v_0_-determination	At 25 °C, 50 mM potassium phosphate buffer, pH 7.5
Free EUGO^†^	80 ± 20	5 ± 1	v_0_-determination	At ambient conditions in 50 mM Glycine·NaOH buffer, pH 8.0
A4E4 complex^†^	40 ± 10	4 ± 1		
A4E4 complex on CS-Az-Bio-NF membrane^† ‡^	170 ± 30	1.1 ± 0.1	Continuous-flow setup	Flow rate of 1 mL/min w/o catalase in main buffer
	350 ± 90	1.2 ± 0.1		Flow rate of 2 mL/min, with catalase in main buffer

^†^These values were determined by testing only one technical replicate at different conditions (*N* = 1) and are thus preliminary

^‡^Amount of EUGO on nanofiber mats was calculated from activity loss of supernatant during loading of samples

**Fig. 5:**
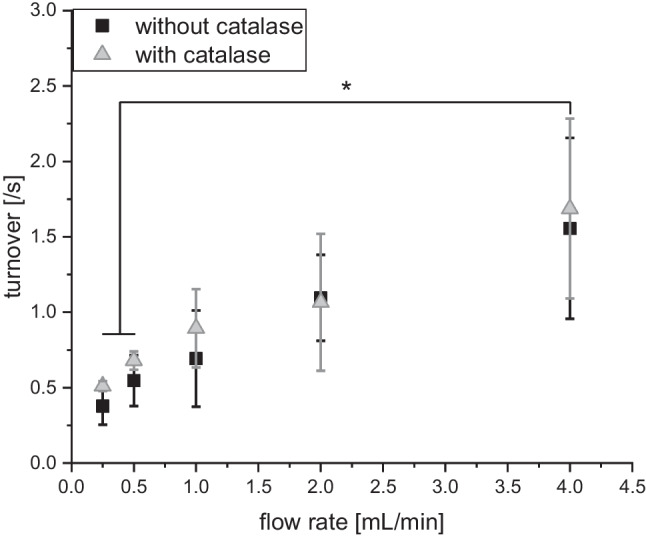
Influence of flow rate on turnover of EUGO, immobilized on CS-Az-Bio-NF, in continuous setup with and without catalase in substrate buffer at excess (2000 μM) vanillyl alcohol (*N* = 3). Null hypothesis testing for zero difference between all samples was performed with ANOVA followed by pairwise comparison with the Tukey-HSD test

With regard to a future application, it is of high interest which flow rate could be tolerated by the fiber mats. Flow rates up to 4 mL/min were tested in our setup without observing detrimental effects such as activity loss or buildup of high back pressure. The area of the CS-NF mesh samples that the flow of the setup passed through was 133 mm^2^. Nevertheless, mechanical properties of the nanofibers were not determined, as no problems with mechanical integrity or degradation were encountered. It was also found that with decreasing flow rates, the product concentration achievable in the downstream flow increased as it was expected for substrate saturating conditions. Thus, the highest flow rate that could be tested with our setup was set by the sensitivity of the product detection and the maximum velocity of the substrate pump to 4 mL/min. Interestingly, the increase in formed product vanillin was less then proportional to the flow-rate reduction, so that turnover decreased significantly with decreasing flow rates, rather than remaining constant as initially expected (Fig. 5). The same trend was also observed when excess catalase was added to the substrate buffer in order to capture H_2_O_2_ developed in the reaction (Fig. 5).

In theory, stacking of multiple fiber mats loaded with catalytically active enzyme should increase the measurable activity linearly. To test the developed CS-Az-Bio-NF on this criterion, three individual reactors were prepared, loaded with EUGO at optimal conditions and subjected to individual flow-rate activity assays. When testing a combination of two of the three reactors connected in sequence (all six possible combinations of reactors were tested), we found that 80 ± 20% of the theoretical maximum activity of EUGO could be measured.

## Discussion

### Recombinant EUGO-StrepTag-II™ forms complexes with StrepTactin™

The observed elution volume of pure EUGO in size exclusion experiments and the resulting estimated molecular weight (130 ± 50 kDa) is in good agreement with the theoretical value of 124 kDa for dimeric EUGO (as estimated using a tool from web.expasy.org/compute_pi/ (Bjellqvist et al. 1994; Bjellqvist et al. 1993; Walker 2005)). The large shift in elution volume and the drastic increase of molecular weight upon incubation of EUGO with StrepTactin™ suggest multimerization between both proteins. The four ligand binding sites of tetrameric StrepTactin™ would, in theory, enable binding of up to four chains of EUGO. A complex of one tetrameric StrepTactin™ with two EUGO dimers (four EUGO monomers and four StrepTactin™ monomers) would thereby result in a 302 kDa complex, which is in the range of the found molecular weight (Fig. 1 a, peak A, 250 ± 80 kDa). The high specific EUGO activity in vanillin assays from fractions collected from this peak signifies that EUGO remains active upon binding to StrepTactin™. Moreover, the fact that EUGO and StrepTactin™ monomers were also found in SDS-PAGE of fractions from peak A (Fig. 1 a and b) also confirmed formation of a stable complex, which is termed A4E4 complex. The observation that complex formation was not hindered upon co-incubation of the protein mixture with an excess of biotin (Fig. 1 a, orange curve) suggests a much stronger interaction of StrepTactin™ with EUGO than with biotin. Determination of *K*_M_ and *k*_cat_ of free EUGO and the A4E4 complex (see Table 2) demonstrated that binding of EUGO with StrepTactin™ did not influence the kinetic properties of EUGO drastically. Depending on the EUGO concentration in the conjugation mixture, higher complexes were also observed (see Fig. S3). Their occurrence can be explained by synergy of the dimerization ability of EUGO and the quaternary structure of StrepTactin™, enabling a multitude of stoichiometries (Nguyen et al. 2016; Jin et al. 2007).

The high elution volume of the second signal (Fig. 1 a, peak D, 22.25 mL) from the mixture of EUGO and StrepTactin™ with a ratio of 1:1 suggests a low molecular weight of < 5 kDa and is in reasonable agreement with that of the single signal found in the chromatogram of free StrepTactin™ (Fig. 1 a, orange curve). Also, results from SDS-PAGE of that signal (Fig. 1 b) suggest the presence of free StrepTactin™. The high elution volume of StrepTactin™ can be explained by interaction of the protein with the column under the chosen conditions. In fact, biotin-saturated StrepTactin™ did not interact with the column and thus, its elution volume shifts into regions actually expected for its size.

In summary, our results clearly demonstrate that simple co-incubation of EUGO and StrepTactin™ in a 1:1 molar ratio efficiently yields A4E4 complex, which fully retains enzymatic activity. Further, the complex multimerization patterns, observed for other ratios, showcase the versatility of the avidin-derived system. We thus expect that at the right conditions, ordered precipitation of large and complex protein lattices on chitosan nanofiber matrices will be feasible and could lead to maximizing catalytic properties of solid-phase biocatalysts in the future.

### CS-Az-Bio-NF mats constitute a water stable immobilization matrix

The results from leaching experiments are well aligned with the previous identified crosslinking optimum (Christ and Menzel 2023) and suggested that only a small portion (~ 2% at irradiation time of > 60 s) of CS-Az-Bio from the dry sample failed to be integrated in the network and thus dissolved during initial washing of the mat. The fact that the mats remained stable and did not release more material after removal of the free polymer chains confirms the high stability of the remaining UV-crosslinked CS-NF mats in aqueous conditions, which was observed before for over 30 days and even under adverse pH values (harsh acidic and basic conditions, 1M HCl and 1 M NaOH) (Christ and Menzel 2023). This is crucial for application as enzyme immobilization matrix.

### A4E4 complex can be immobilized on CS-Az-Bio-NF mats by biotin-affinity binding

By incubation of prewashed but otherwise untreated nanofiber samples from CS-Az and CS-Az-Bio, it could be established that both sample types were able to immobilize significant amounts of A4E4 complex. The fact that only a small difference of 33% greater catalytic activity of biotinylated CS-Az-Bio-NF samples in respect to nonbiotinylated CS-Az-NF was found was attributed to an overshadowing effect of nonspecific binding. These are hypothesized to stem from electrostatic interactions of the positively charged chitosan nanofiber matrix with negatively charged proteins. Respective pI-values were estimated (using a tool from web.expasy.org/compute_pi/) (Bjellqvist et al. 1994; Bjellqvist et al. 1993; Walker 2005) to be 9 for StrepTactin™ and 5 for EUGO; thus, a positive charge is expected for StrepTactin™ and a negative charge is expected for EUGO under the chosen alkaline (pH 8.0) conditions. Blocking experiments finally demonstrated that most of the protein binding ability of CS-Az-Bio-NF is highly specific to the biotinyl-A4E4 complex interactions. Nevertheless, in untreated samples, this effect can be overshadowed by unspecific interactions. Washing experiments further indicated a high protein retention ability of CS nanofibers even under adverse conditions. The considerably higher decrease in relative activity of CS-Az-Bio-NF compared to CS-Az-NF was attributed to breaking of specific interactions at high salt concentrations. Interestingly, the portion of EUGO activity from CS-Az-Bio-NF, that seemed to be bound via unspecific interactions, resisted release under the tested buffer conditions.

It is noteworthy that the reduction of A4E4 complex concentration during following immobilization incubations did not significantly decrease the load of CS-Az-Bio-NF mats, but decreased that of CS-Az-NF mats (see Table 1). This further indicates a lower dissociation constant of CS-Az-Bio-NF, which is expected when comparing specific protein-ligand binding with nonspecific immobilization. Based on our measurements, the maximum capacity of CS-Az-Bio-NF seems to be close to 10 μg EUGO per milligram (assuming unaltered enzyme kinetics, Table 2) which is equivalent to an EUGO activity of 20 μM/min/mg fiber sample under the chosen conditions. Based on the multimeric structures of tetrameric StrepTactin™ and dimeric EUGO, it seems reasonable to assume that the A4E4 complex would cover a circular surface area of about 60 nm^2^. The average fiber diameter of ~ 300 nm (Christ and Menzel 2023) can be used to calculate the surface to volume ratio of 1.3·10^7^ (m^2^/m^3^) of the CS-NF. With an assumed density of 1 g/cm^3^ of the dry fibers, a surface of 13 m^2^ per gram dry mass can be estimated. These values can be combined to estimate a theoretical maximum load of 352 nmol/g which in turn results in 50 μg/mg fibers. This estimation is in line with the above experimentally determined maximum capacity of the fibers under our tested conditions. One key learning from the theoretical estimation for the maximum enzyme capacity of our fibers is that CS-NF can be fully loaded under the right conditions and thus, the nanofiber approach seems worthwhile. We furthermore expect that by using nanofibers with diameters below 100 nm, the maximum capacity could be drastically increased in future applications. Altogether, the results show that sufficient amounts of EUGO can be immobilized and retained effectively on CS-Az-Bio-NF mats via specific affinity interactions.

### Photocrosslinking process can affect affinity binding activity of chitosan nanofibers

The finding that irradiation of CS-Az-Bio-NF with UV light was associated with a significant reduction of their ability to remove EUGO activity from the supernatant indicates a destructive effect of UV irradiation on the affinity immobilization capacity of the samples. UV treatment of CS-Az-Bio-NF mats for extended time periods (240 min, 360 J/cm^2^) resulted in a similarly low immobilized EUGO activity as nonbiotinylated CS-Az-NF mats (Table 1). It seems possible that the remaining activity was entirely due to nonspecific binding, thus indicating complete degradation of biotin. Samples that were treated for shorter times (3 min, 18 J/cm^2^) exhibited a significantly higher activity loss from the supernatant and a significantly higher immobilized EUGO activity. It must also be mentioned that noncrosslinked (0 min) CS-Az-Bio-NF mats acquired a gel-like and partially dissolved state upon contact with buffer while achieving the highest EUGO activity loss from supernatant but significantly lower observable immobilized activity. Perhaps, loss of nanofiber form and resulting gel consistency reduced the substrate accessibility of bound EUGO in these cases. Another finding worth discussing is the discrepancy between adsorbed EUGO on fibers determined from supernatant loss in contrast to the values that were determined directly from EUGO activity on fibers. These findings indicate a reduction of enzyme activity during immobilization of EUGO on the fibers. We hypothesize that binding of the A4E4 complex to the fiber mats reduced the EUGO activity due to substrate inaccessibility or immobilization induced conformational changes to the protein. It seems that the approach of pre-electrospinning biotinylation, used by us, creates the need to compromise between an efficient immobilization and sufficient crosslinking. This may be avoided by employing alternative methods, e.g., post-crosslinking biotinylation or the use of monochromatic irradiation at a wavelength which excites the aryl azide but does not damage the biotin moiety of modified CS mats. As previous reports demonstrate (Christ et al. 2020; Christ and Menzel 2023), water-stable mats can be achieved with as little as 1-min irradiation time. Thus, we advise future investigation to minimize the applied total radiation to a necessary minimum.

### CS-Az-Bio-NF mats can be applied in a continuous-flow setup while conserving enzyme kinetics

**Fig. 6:**
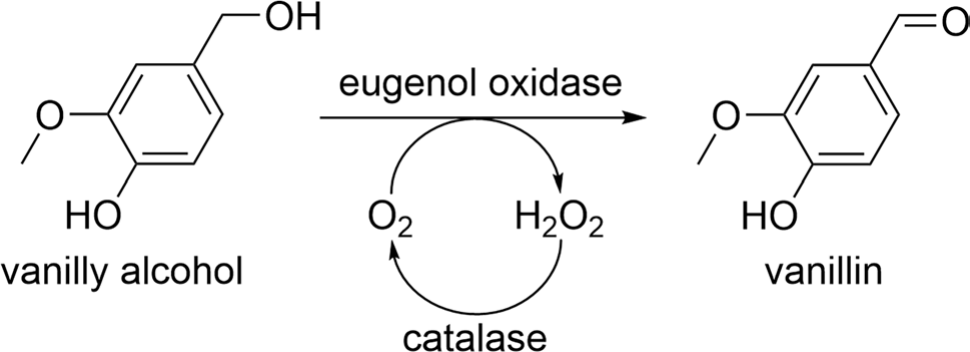
Oxidation of vanillyl alcohol to vanillin, catalyzed via EUGO, as used for activity determination in vanillin assay. Replenishment reaction of oxygen via catalase is also included

The finding that over 93% of the initially measured activity of fiber mats could be observed when the exact samples were tested in our continuous-flow setup demonstrates that photocrosslinked CS-Az-Bio-NF can be successfully used in a continuous mode as immobilization matrix for at least the A4E4 complex. The fact that no reduction in EUGO activity and no significant loss of the enzyme over time were observed in the continuous-flow experiments is further evidence of strong protein immobilization. Regarding the enzyme kinetics, it was found that the EUGO preparation used in our study was close to that reported in the literature (see Table 2). The difference in turnover number (*k*_cat_) might be caused by the use of slightly different reaction parameters (i.e., temperature, pH, buffer composition). Moreover, binding to StrepTactin™ did not alter catalytic parameters drastically (Table 2). The increase of *K*_M_ for immobilized EUGO during increase of the flow rate from 1 to 2 mL/min, suggests that substrate binding is hindered under continuous-flow conditions. We suspect a steric conflict between fibers and enzymes as the reason for this. Another possible explanation could be insufficient amount of substrate in the assay which could reduce turnover numbers. However, even at the lowest flow rate tested (0.25 mL/min), the substrate concentration was reduced by only 20 μM. This value is small compared to the total substrate concentration of 2000 μM. Thus, insufficient substrate saturation of the enzyme is not a valid explanation for the observed reduction in turnover (cf. *k*_cat_, Table 2) and can be excluded. Another possible explanation to discuss would be that low flow rates increase the concentration of H_2_O_2_, the second product of the reaction (Fig. 6), which could potentially damage and inactivate EUGO.

However, the same trend was also observed when excess catalase was added to the substrate buffer in order to capture H_2_O_2_ developed in the reaction (see Fig. 5). Thus, enzyme inactivation by peroxide cannot explain the flow-rate dependency of turnover either. As our setup did not allow for a control of oxygen concentration, we were not able to test the effect of this second substrate on the kinetic properties. Assuming a concentration of dissolved oxygen in the substrate buffer of up to 9.1 mg/L (Bozorg-Haddad 2021), a reduction of oxygen concentration by just 4% (0.51 mM → 0.49 mM) would be expected at a flow rate of 0.25 mL/min. While EUGO oxygen-related kinetics are currently not available, a *K*_M_ (O_2_) of 24 μM has been reported for VAO (Fraaije and van Berkel 1997). With catalase addition, an increased effective oxygen concentration is expected. On this basis, we also exclude oxygen limitation as explanation for the observed flow-rate dependency. Further research is necessary to find an explanation for this unexpected behavior.

The fact that stacking of fiber mats clearly resulted in additive properties indicates excellent applicability in various areas. We thus conclude that enzyme-loaded CS-NF mats are suitable for application in continuous-flow setups, as they also maintain their mechanical integrity and catalytic properties under at least moderately high flow rates. Even though mechanical properties were not determined in our case, our subjective experiences of macroscopic handling of the nanofiber CS-Az-Bio-NF and CS-Az-NF samples were in line with previously determined tensile strengths of comparable chitosan-based nanofibers being 14 MPa and 5 MPa in dry and wet state (Li et al. 2015). Further optimization of especially the crosslinking density and conditions is expected to further increase these values and lead to fibers that could be used for industrial processes. The kinetic properties of EUGO show some dependency on the flow conditions, but not to a degree that would obstruct industrial application. Further, we demonstrated that accurate kinetic measurements of the immobilized enzyme can easily be undertaken using a flow setup. This will greatly benefit the optimization of preparative processes involving CS-NF-based biocatalysts and outlines possible analytical applications of the concept. Also, multi-enzyme cascades can become possible as the system can potentially be used for all suitable catalytic enzymes equipped with a StrepTag-II™ or biotin groups.

In conclusion, this report demonstrates that nanofiber meshes based on chitosan, modified with arylazides and biotin (CS-Az-Bio-NF), electrospun, and photocrosslinked employing our previously published methods, are able to retain StrepTactin™-containing protein complexes in a biotinyl-specific manner. Only a minor decrease of enzymatic activity was observed upon immobilization, strongly indicating that no irreversible denaturation of enzyme occurred. While the protein capacity per volume is comparable to that of commercially available conventional immobilization matrices (e.g., Sepharose gels or beads), biotinylated CS-NF have the advantages of electrospun nanofiber meshes, such as high porosity, high internal surface, and decent mechanical sturdiness. This was herein demonstrated, as the material tolerated moderately high flow rates of up to 0.24 L/h (4 mL/min passing through 133 mm^2^ of CS-NF mesh) in a continuous-flow setup. This promises improvements for existing applications of protein immobilization and potentially enables entirely new applications in the future.

### Supplementary information

ESM 1 (PDF 520 kb)

## Data Availability

The datasets generated and analyzed during the current study are available from the corresponding author upon reasonable request.
