# Supplementary material for: Application of electrospun chitosan-based nanofibers as immobilization matrix for biomolecules
Source: Appl Microbiol Biotechnol. 2023 Sep 27;107(23):7071–87. doi: 10.1007/s00253-023-12777-w (PMC10638201; doi:10.1007/s00253-023-12777-w)
Supplement: Supplementary file 1 — (PDF 520 kb) [file 253_2023_12777_MOESM1_ESM.pdf]

Applied Microbiology and Biotechnology

Application of electrospun chitosan nanofibers as immobilization matrix for biomolecules

Henrik-Alexander Christ[a], Nils Peter Daniel[b], Jennifer Solarczek[b], Leonard Sebastian Fresenborg[c], Anett Schallmey[b], Henning Menzel[a,].*

[a] Braunschweig University of Technology, Institute for Technical Chemistry, Hagenring 30, 38106 Braunschweig, Germany.

[b] Braunschweig University of Technology, Institute for Biochemistry, Spielmannstraße 7, 38106 Braunschweig, Germany.

[c] Goethe University, Department of Molecular Cell Biology of Plants, Max-von-Laue-Str. 9, 60438 Frankfurt am Main, Germany.

* Corresponding authors e-mail address: h.menzel@tu-braunschweig.de

Supplementary information:

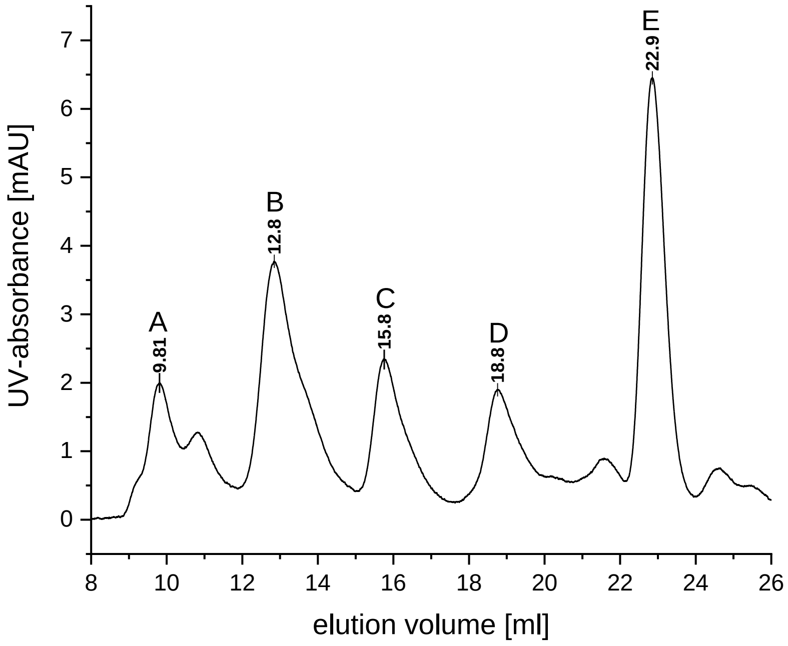

Fig. S1 Separation of Protein Standard Mix 69385 on Superdex 200 increase 10/300 GL column at a flowrate of 0.5 mL/min using detection signal at 280 nm. Marked peaks with letters represent the four proteins: bovine thyroglobuline (A, 9.81 mL, 670.0 kDa), y-globulines from bovine blood (B, 12.8 mL, 150.0 kDa), albumin chicken egg grade VI (C, 15.8 mL, 44.3 kDa), ribonuclease A-type I-A from bovine pancreas (D, 18.8 mL, 13.7 kDa) as well as low molecular weight marker: para-aminobenzoic acid (E, 22.9 mL, 0.14 kDa)

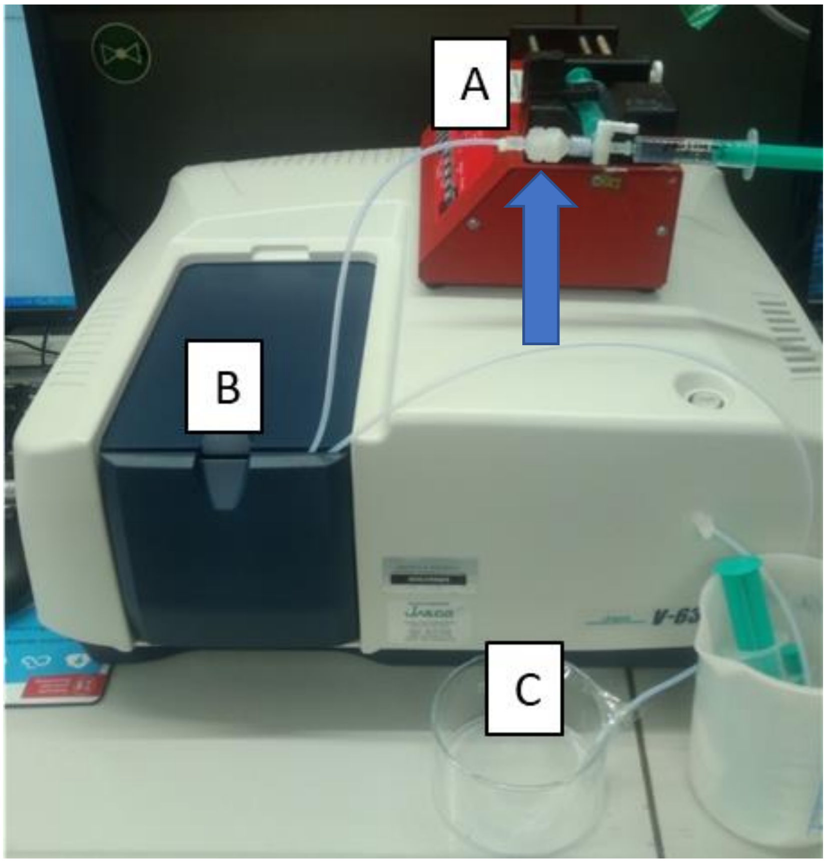

Fig. S2 Photograph of experimental setup for all continuous flow experiments: A: syringe pump LA-30 with 20 mL single-use syringe, gate and reusable syringe filter holding the actual fiber sample with immobilized proteins (see blue arrow), B: UV-VIS spectrophotometer V-630 with flow-through cuvette, connected to reactor, C: liquid waste container

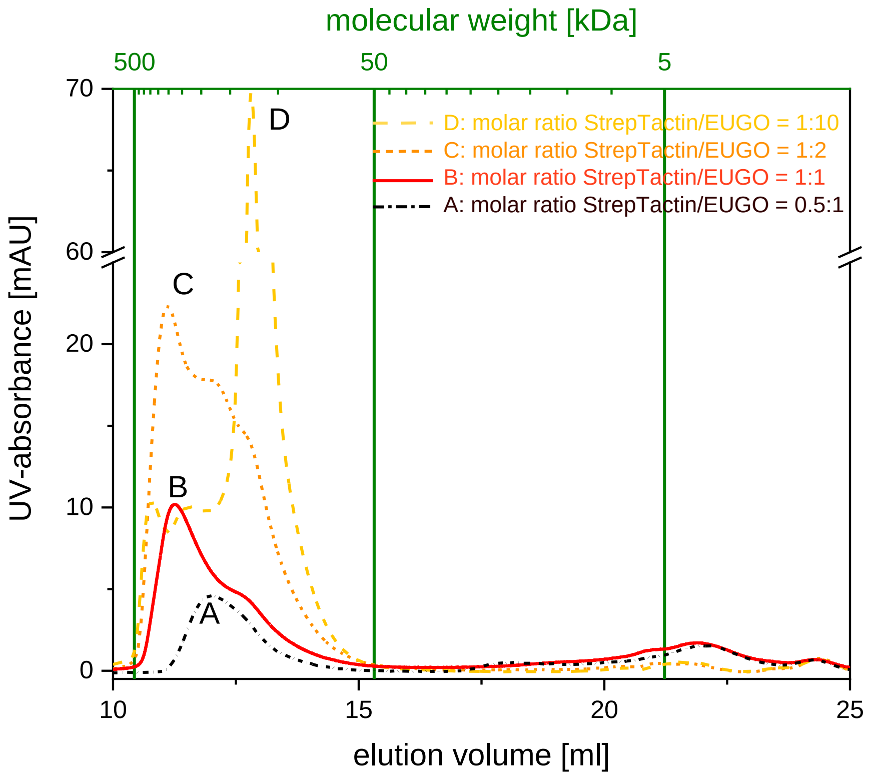

Fig. S3 Stacked elugrams of EUGO and StrepTactin™ incubated together for 18 h in mixtures at different ratios (StrepTactin/EUGO: (A = 0.5:1; B = 1:1; C = 1:2; B = 1:10), forming various complexes together

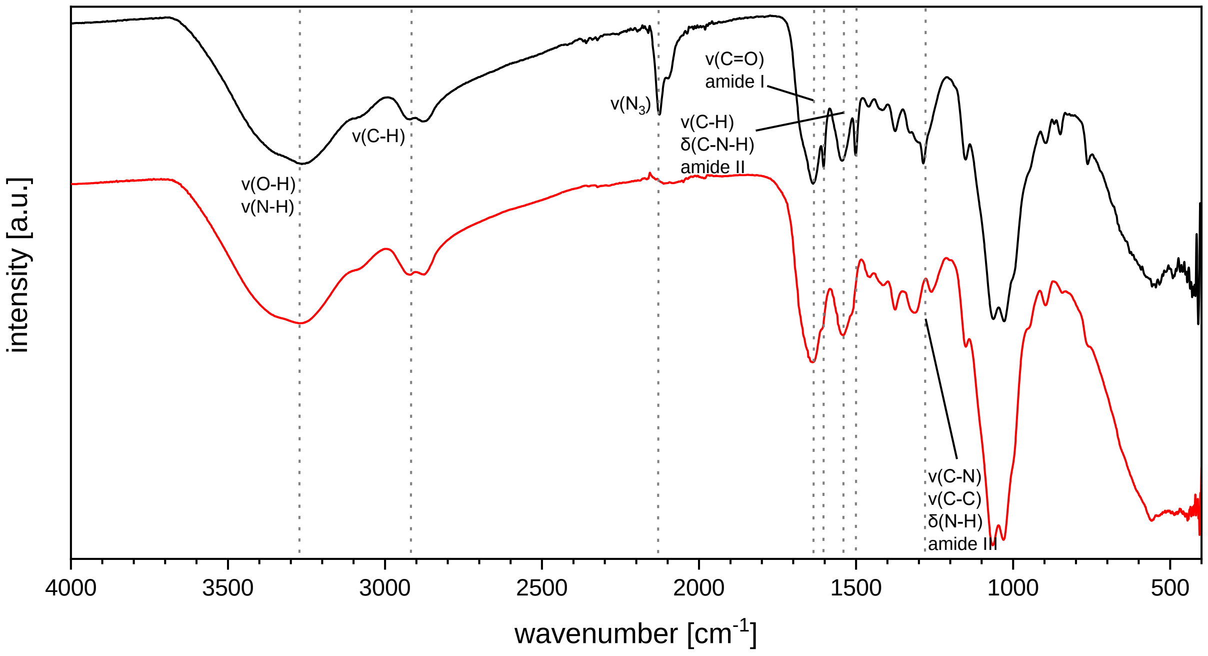

Fig. S4 Exemplary ATR-IR spectra of the used CS-Az-Bio derivative (black curve) as well as all its nanofibrous electrospun and photocrosslinked CS-Az-Bio-NF form (red curve). The spectrum of CS-Az-Bio derivative was already shown before. (Christ H-A, Menzel H (2023) Electrospinning and Photocrosslinking of Highly Modified Fungal Chitosan. Macromol. Mater. Eng. 308:2200430. doi: 10.1002/mame.202200430)

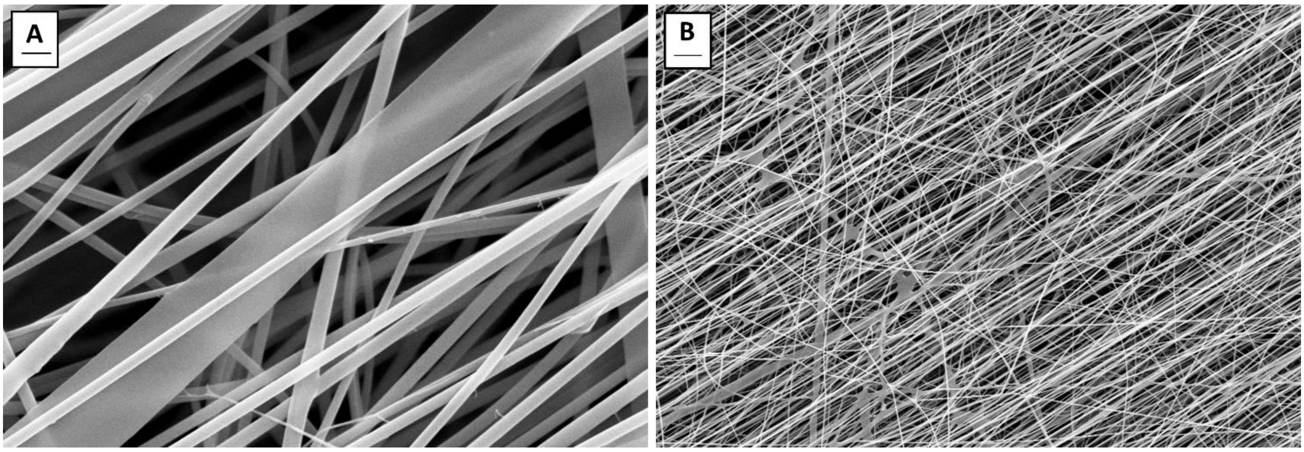

Fig. S5 Representative SEM images at 5.000x (A) and 500x (B) magnification of CS-Az-Bio-NF used for immobilization of proteins. Insets represent 1µm (A) and 10 µm (B)

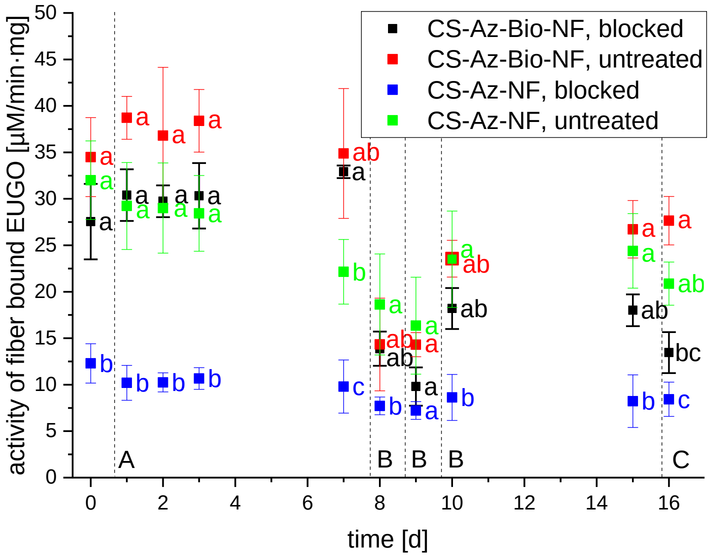

Fig. S6 Original data of Figure 3, analyzed with ANOVA (α = 0.05) followed by pairwise comparison was done with TUKEY-HSD test, demonstrating that the untreated CS-Az-NF meshes showed significantly lower activity in buffer A compared to both the biotinylated and blocked samples. Letter code from a-c was used to group data from each experimental day into categories with significant differences. All fiber samples were measured simultaneously during each experimental day, thereby insuring low systematic errors withing such sets of data. Comparison of data sets between individual points of time (e.g., experimental days) are not shown, as higher systematic errors are expected.
